# Classification of breast lesions in ultrasound images using deep convolutional neural networks: transfer learning versus automatic architecture design

**DOI:** 10.1007/s11517-023-02922-y

**Published:** 2023-09-22

**Authors:** Alaa AlZoubi, Feng Lu, Yicheng Zhu, Tao Ying, Mohmmed Ahmed, Hongbo Du

**Affiliations:** 1 School of Computing and Engineering, University of Derby, Derby, DE22 3AW UK; 2 Department of Ultrasound, Shuguang Hospital affiliated to Shanghai University of Traditional Chinese Medicine, Shanghai, China; 3 Department of Ultrasound, Pudong New Area People’s Hospital affiliated to Shanghai University of Medicine and Health Sciences, Shanghai, 201200 China; 4 Department of Ultrasound, Sixth People’s Hospital, Shanghai, China; 5 School of Computing, The University of Buckingham, Buckingham, MK18 1EG UK

**Keywords:** Breast cancer, Ultrasonography, Cancer recognition, Deep convolutional neural network, Transfer learning, Automatic architecture design, Bayesian optimization

## Abstract

Deep convolutional neural networks (DCNNs) have demonstrated promising performance in classifying breast lesions in 2D ultrasound (US) images. Exiting approaches typically use pre-trained models based on architectures designed for natural images with transfer learning. Fewer attempts have been made to design customized architectures specifically for this purpose. This paper presents a comprehensive evaluation on transfer learning based solutions and automatically designed networks, analyzing the accuracy and robustness of different recognition models in three folds. First, we develop six different DCNN models (BNet, GNet, SqNet, DsNet, RsNet, IncReNet) based on transfer learning. Second, we adapt the Bayesian optimization method to optimize a CNN network (BONet) for classifying breast lesions. A retrospective dataset of 3034 US images collected from various hospitals is then used for evaluation. Extensive tests show that the BONet outperforms other models, exhibiting higher accuracy (83.33%), lower generalization gap (1.85%), shorter training time (66 min), and less model complexity (approximately 0.5 million weight parameters). We also compare the diagnostic performance of all models against that by three experienced radiologists. Finally, we explore the use of saliency maps to explain the classification decisions made by different models. Our investigation shows that saliency maps can assist in comprehending the classification decisions.

**Graphical Abstract:**

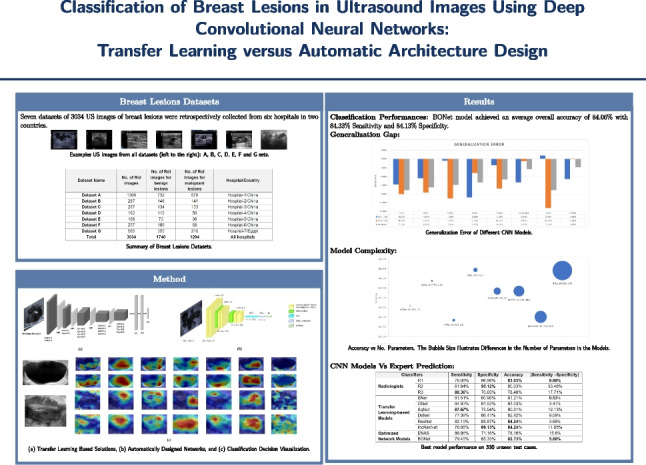

## Introduction

Breast cancer is one the most common cancers, accounting for 30% of all cancers found in females [1]. Early detection is critical for successful treatments. Ultrasound (US) scan is an image modality widely used for breast lesion examination due to its non-invasiveness, lack of radiation, and low-cost. However, reading and interpreting US images face specific challenges. First, the images are adversely affected by speckle noises and artifacts, making images blurry and of poor contrast. Second, image quality and appearance tend to vary across US machines of different makes. Both issues contribute to possible inter- and intra-observer variability among radiologists when describing malignancy features. Computer-aided diagnosis (CAD) systems aim to produce robust and accurate models for classifying US images of breast lesions, reduce the burden on the radiologists by expediting the examination process, and provide a second opinion on the lesion statuses especially for borderline cases. Consequently, CAD systems have attracted a substantial amount of research interest.

Deep convolutional neural networks (DCNNs) have demonstrated outstanding performance in object recognition in natural images across various application domains due to their capabilities of learning discriminative features from the images automatically [2]. In recent years, efforts in adapting and developing DCNN solutions for breast lesion classification in US images have been intensified [3–12]. Although high levels of test accuracy on datasets of different sizes have been reported, there is lack of systematic and thorough evaluations on main performance indicators conducted on a common large-scale dataset across the existing solutions.

This paper presents a comprehensive evaluation of transfer learning based CNN models and automatically designed CNN models for breast lesion classification in US images. The evaluation uses seven datasets totaling 3034 US images of single and multiple lesions acquired from machines of different makes in several hospitals in two countries. The work is motivated by the interest in a thorough comparison of the different CNN design approaches. The paper particularly aims to make the following contributions. First, six well-known CNN architectures are adapted to train DCNN models (BNet, GNet, SqNet, DsNet, RsNet, IncReNet). Second, Bayesian optimization is employed to automatically design a CNN architecture along with its modeling hyperparameters (BONet). The paper then conducts a comparative analysis of performances of all network models in terms of accuracy (overall accuracy, sensitivity, and specificity), robustness (generalization gaps of accuracies between internal and external tests), and model complexity (the total number of weights). Besides, the performances of all network models are then compared with those of experienced radiologists. Two visualization methods, EGrad-CAM and Ablation-CAM, are further used to highlight image regions to gain better understanding about the decisions made by the different CNN models.

## Related work

There are mainly two approaches for designing DCNN solutions: (a) building and training a network from scratch through either handcrafted or automatic/semi-automatic design and (b) adapting a pre-trained network models. For the handcrafted design, many attempts have been made to determine DCNN’s structural hyperparameters (e.g., number of layers, number of filters, operations, types of layers, layer connectivity) and training hyperparameters (e.g., initial learning rates, number of epochs, optimization method). Examples of known handcrafted DCNNs for natural images include AlexNet [2], VGGNet [13], GoogleNet [14], ResNet [15], Inception [16], DenseNet [17], and SqueezeNet [18]. However, manual design of architectures specifically for breast lesion classification in US images remains limited [9, 10]. For instance, the Fus2Net architecture consists of three basic convolutional layers with various numbers of 3 × 3 filters, followed by two blocks of modules with different combinations of convolutional and pooling operations to fuse low and high dimensional features [10]. Handcrafted designs require in-depth knowledge in DCNN with a degree of trial and error. Recently, there is a growing interest in automatically optimizing DCNNs. Examples include neural architecture search (NAS) [19] and efficient neural architecture search (ENAS) [20]. The ENAS approach uses a recurrent neural network (RNN) as the controller and reinforcement learning (RL) as the search strategy to search for optimal CNN architectures. ENAS has shown its potentials for breast lesion classification in US images with overall accuracies of 85.8%, 82.7%, and 88.1% respectively for an internal test and two external tests out of 2167 US images [11, 12].

The adaptation approach for breast lesion classification in US images, as well as other applications, using transfer learning from pre-trained models on the ImageNet dataset [21], appears more popular [3, 22]. This approach addresses the issue of lack of quality medical images and saves computation time by refining an existing network model. Byra et al. [5] adapted and trained a VGG19 model to classify breast lesions in US images. A dataset of 882 images (204 malignant and 678 benign) was used, and the model achieved an AUC of 93.6%. Huynh et al. [6] used AlexNet [2] as the pre-trained model to extract features to train a support vector machine. A dataset of 1125 cases were used to tune and test the model, achieving an AUC of 88%. Byra et al. [7] further adapted InceptionV3 and compared with VGG19. The OASBUD [23] dataset of 100 US images (48 benign and 52 malignant) was used to train and test the models. The InceptionV3 model achieved an accuracy of 78% (77% sensitivity, 78% specificity, and 85.7% AUC) whereas the VGG19 model achieved an accuracy of 82% (70% sensitivity, 78% specificity, and AUC of 82.2%). Hijab et al. [8] used transfer learning to fine-tune VGG16 for classifying breast lesions using a dataset of 1300 US images. The refined model achieved an accuracy of 97% and an AUC of 98%. Recently, a generic CNN model based on VGG19 architecture with transfer learning for classifying breast and thyroid lesions was proposed [3]. Singular value decomposition (SVD) was used to augment data from 719 thyroid images (421 benign and 298 malignant) and 672 breast images (373 benign and 299 malignant) obtained from US machines of different makes. Average accuracy of 86.5% for the thyroid model and 89% for the breast model were reported.

## Materials and methods

### Data acquisition, annotation, and pre-processing

**Fig. 1:**
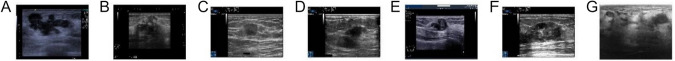
Examples US images from all datasets (left to the right): **A**, **B**, **C**, **D**, **E**, **F** and **G** sets

In this study, seven datasets of US images of breast lesions retrospectively collected from six hospitals in two countries were used: six datasets were collected from different hospitals in China, and the seventh is a public domain dataset (BUSI) collected from a hospital in Egypt [23]. All gray-scale US examinations were performed in those hospitals using US machines of different makes and models including Siemens Oxana 2, Siemens S3000, Toshiba Apolio 500, GE, Logic E9, and Philips Epic 7. Each original ultrasound image may contain regions showing one or two lesions in the same breast mass. Table 1 provides a summary of the datasets. Figure 1 shows one selected example image from each dataset. Qualified radiologists, each with 10 to 25 years of experience, used the software tool reported in [3] to identify a lesion region in the image and cropped the region manually by placing coordinate points on the lesion boundary in datasets A–F. Using these points, the software automatically obtained a rectangular bounding box defining a minimum-area-rectangle *R* that contained the lesion. Manual cropping is inherently imprecise, and the number of points placed on the lesion boundary also varies between images. To ensure the inclusion of the whole lesion and the contrast information in surrounding areas useful for the lesion classification [3, 24], approximately 8% of the width and height of *R* is added to the margin, producing a new rectangle *R’*, referred to as the region of interest (RoI). The type of the lesion in each RoI image (benign or malignant) was confirmed through histopathological assessment of tissue samples and served as the ground-truth.

**Table 1:** Summary of breast lesions datasets

Dataset name	No. of RoI images	No. of RoI images for benign lesions	No. of RoI images for malignant lesions	Hospital/country
**Dataset A**	1308	732	576	Hospital-1/China
**Dataset B**	287	146	141	Hospital-2/China
**Dataset C**	287	134	153	Hospital-3/China
**Dataset D**	162	112	50	Hospital-4/China
**Dataset E**	168	72	96	Hospital-5/China
**Dataset F**	257	189	68	Hospital-6/China
**Dataset G**	565	355	210	Hospital-7/Egypt
**Total**	**3034**	**1740**	**1294**	**All hospitals**

Besides, three radiologists with 10 to 25 years of experience between them observed all 330 US images in datasets D and E and labeled them as benign or malignant without referring to any other patient information (e.g., blood test) or the pathology report. We later use those labels and the predicted labels by the CNN models to compare against the ground-truth. Since both pre-trained models and optimized network models require input images of a fixed size, the cropped RoI images were resized to the desired size required by the input layer of a specific network.

For lesion recognition from ultrasound images, it is particularly challenging to gather and annotate large-scale datasets from multiple medical centers. On the other hand, training robust CNN models of complex structures with many weight parameters do require many training examples. Data augmentation therefore becomes essential to overcome the data limitation by expanding the number of training examples. In this study, both for adapting the pre-trained networks and designing the CNN architecture from scratch, the mirroring and the singular value decomposition methods described in [3] were employed. The mirroring method creates a duplicate image by flipping the image across its vertical axis. The SVD method preserves the geometric shapes in the image and generates approximate images with different levels of compression. For each RoI image, one additional image was generated by mirroring and three additional images by SVD with ratios of the selected top singular values set at 25%, 35%, and 45%.

### Transfer learning based approach for lesion classification

The parameters of the CNN models VGG19 [13], GoogleNet [14], Resnet101 [15], Inception-ResNet-v2 [16], DenseNet [17], and SqueezeNet [18] were first pre-trained on the ImageNet dataset [21] for object recognition. Following the first attempt in DCNN models by AlexNet in 2012 [2], VGG [13] was developed as the first architecture where all convolutional layers are 3 × 3 stride 2, all pooling layers are 2 × 2 max-pooling with stride 2, and the number of channels is doubled after the pooling layer. VGG was followed by GoogleNet [14] which focuses on model efficiency by reducing the number of parameters, memory usage, and computation time. Besides, the concept of inception as a local structure repeats many times throughout the network. Between 2014 and 2015, the idea of batch normalization was developed and adopted by an innovative architecture Resnet101 [15]. The number of DCNN layers has also increased from 22 to 152 in that period. In 2017, the densely connected neural networks where each layer is connected to every other layer in a feedforward fashion were proposed [17]. This approach can be seen as a way of maximizing skip connections, bringing extracted features from all layers to the final decision. On the other hand, several attempts have been made to develop efficient and small size networks such as SqueezeNet [18] for medical diagnostics [25]. In this study, these networks were selected due to variations in their depths (18–201 layers), numbers of parameters (1.24–144 millions), and network topological structures. The layers trained using the CNNs [13–18] on the ImageNet dataset [21] were further adapted for cancer recognition task. For a systematic and fair comparison, we set the network parameters for all models as follows: 120 epochs with iteration number = 14,280, initial learn rate = 0.0001, and mini-batch size = 64. The other parameters were set as default values of each network.

#### BNet—VGG19 transfer learning

Inspired by the DCNN architecture used in [3], we adopted the same architectural parameter settings to train the model BNet. VGG19 [13] has 47 layers (16 convolutional layers, 3 fully connected layers) and approximately 144 million weight parameters. Each convolutional layer consists of various kernels of size 3 × 3. The architecture of the CNN model was adapted by replacing and fine-tuning the last fully connected layer and the softmax layer. Since the images have binary class labels, the last fully connected layer in the original architecture was replaced by a new fully connected layer for binary classification. The last “Dropout” layer for BNet was set to 25%. The network has an input image size of 224 × 224 × 3.

#### GNet—Google transfer learning

We adopted the GoogleNet CNN [14] and train the model GNet. The architecture [14] has 9 inception modules of 22 layers deep (27 including the pooling layers) with a GAP layer at the end of the last inception module. It has approximately 7 million weight parameters. The architecture [14] was adapted by replacing and fine-tuning the last fully connected layer and the softmax layer. The last fully connected layer was also replaced by a new fully connected layer for two classes. The network has an input image size of 224 × 224 × 3.

#### DsNet—DenseNet transfer learning

We adopted the CNN [17] that uses the connectivity pattern where the input to the next layer is the concatenation of all the previous layers inputs. In other words, DenseNet has a loop connectivity pattern where each layer receives signals from all its preceding layers and the input combined by channel wise concatenation. DenseNet [17] has 201 layers with 20 million parameters and uses multiple dense boxes and pooling layers. Similar to GNet, the architecture was adopted by replacing and fine-tuning the last fully connected layer and the softmax layer. A new fully connected layer of two classes replaces the last fully connected layer in the original architecture. The network also has an input image size of 224 × 224 × 3.

#### RNet—ResNet 101

ResNet 101 [15] is a residual learning framework to facilitate the training of deep networks and contains 101 layers with 44.5 million parameters. It can be seen as an extension of the skip connections in ResNet by skipping ahead two layers rather than one like the normal residual building block. The architecture of was adapted by replacing and fine-tuning the last fully connected layer and the softmax layer. A new fully connected layer of two classes replaced the last fully connected layer in the original architecture. The network also has an input image size of 224 × 224 × 3.

#### IncResNet—Inception-ResNet-v2

Inception-ResNet-v2 [16] uses a set of inception modules and incorporates residual connections. It replaces the filter concatenation stage of the inception architecture. The network has 164 layers with 55.9 million parameters and an input image size of 299 × 299 × 3. Similar to RNet, we adopted the CNN [16] by replacing and fine-tuning the last fully connected layer and the softmax layer. A new fully connected layer of two classes replaced the last fully connected layer in the original architectures.

#### SqNet—SqueezeNet transfer learning

All aforementioned networks are complex with many weight parameters. Therefore, we purposely adopted one CNN that has relatively fewer parameters than others. SqueezeNet [18] uses 1 × 1 convolutional layers, decreases the number of input channels to 3 × 3 filters, and down-samples later in the network. It consists of 2 convolutional layers, 8 fire modules, max pooling layers, and a GAP layer and has approximately only 1.24 million parameters. The architecture [18] was adapted by replacing and fine-tuning the last 1 × 1 convolutional layer and the classification prediction output of two classes. The network has an input image size of 227 × 227 × 3.

### Automatic architecture design for lesion classification

Automatic architecture search often involves training one neural network to optimize the architecture of another neural network, but this approach of designing CNN architectures requires computational time and powerful hardware. This section presents our method of searching and designing an optimized CNN architecture. The proposed method performs two consecutive tasks: (i) searching for an optimal CNN architecture and hyperparameters using Bayesian optimization tailored for breast lesion US images and (ii) training the CNN model to classify breast lesions. For the first task, we start by defining a backbone CNN architecture and search spaces for architectural and training hyperparameters. This is then followed by employing Bayesian optimization to search for the optimal network (optimal architecture and training parameters) for accurate classification of breast lesions in US images. More details are given as follows.

#### CNN backbone architecture and hyperparameter search space

In principle, a CNN architecture consists of sequential blocks each of which contains convolutional layer, normalization, activation, and pooling. Deeper convolutional layers may have larger numbers of kernels. A global average pooling (GPA) layer may exist at the end of the final block to reduce the feature dimensionality. Therefore, our proposed backbone architecture consists of an input layer of size 128 × 128, three blocks each of which contains convolutional layer using 3 × 3 kernels, batch normalization and Relu activation, max-pooling with size 2 × 2 and stride 2 at the end of each block except the last one, GPA layer after the final block, fully connected layer, and softmax followed by classification output layer with two classes.

When designing a CNN model, many hyperparameters, e.g., the number of epochs, mini-batch size, regularization, solver, and initial learn rate, are involved and crucial for the model performance. Including all these hyperparameters for optimization will increase the search space exponentially which in turn drastically increases the costs of computation. Therefore, we defined a search space of five optimizable hyperparameters as follows: the number of convolutional layers in each block *B*: [1, 5], solver *Opt*: {adam, sgdm, rmsprop}, L2 regularization *Lr*: [1e − 10, 1e − 2], mini-batch size *Mn*: {32, 64, 128}, and maximum number of epochs *Epo*: [50, 250]. The number of kernels in the first block is given in Eq. 1 and doubled in the second and third blocks.

1 $$\mathrm{numKernal}=\frac{128}{\sqrt{\mathrm{depth}}}$$

where 128 is defined empirically to ensure sufficient number of kernels in the first block when the depth (i.e., the number of blocks in the architecture) is maximum. The $\mathrm{numKernal}$ is rounded towards nearest integer. Equation 1 ensures reasonable number of parameters when the depth increases. The other network parameters were set as follows: convolutional stride = 2, padding = 1, max-pooling stride = 2, initial learn rate = 0.0001, momentum = 0.9.

#### CNN architecture search using Bayesian optimization

Given the backbone architecture and the search space as described in the previous section, we used Bayesian optimization to tune the hyperparameters of the network. The set of architectural and training parameters to be optimized is defined as *hp*^(*i*)^ = {*B*, *Opt*, *Lr*, *Mn*, *Epo*} where *hp*^(*i*)^ is the hyperparameters setting at *i*th search iteration. The objective function *f*(*hp*) is set as the classification error (CaError) on a set *V* of validation examples when modeling the backbone architecture with the setting *hp*.

Bayesian optimizer requires defining initial points to build the surrogate model, and hence we set the number of seed points to 4; i.e., we select randomly 4 hyperparameters settings (*hp*) to evaluate the objective function prior using the acquisition function. We searched for 50 architectures and select the optimal one. The following steps outline the search strategy:

- Step 1: Bayesian optimizer randomly selects 4 of *hp* hyperparameters settings and model the backbone architecture as defined early in this section.
- Step 2: Gaussian process model (regression) is used to create a surrogate model *G*(*hp*) using the classification errors produced when modeling the backbone architecture with the 4 hyperparameters settings.
- Step 3: Using the output surrogate model defined in step 2, the expected improvement *E*(*hp*) method is used to select a new hyperparameter setting *hp’*. The *E*(*hp*) acquisition function selects *hp’* as the one that has the highest expected improvement over the current best observed point of the objective function.
  $$E(hp) = E(max({f}{\prime}(hp) - G(hp)), 0)$$

where *G*(*hp*) is the current posterior distribution of the surrogate model and *f’*(*hp*) is the best observed point (or setting) of the objective function at this iteration of the search.

- Step 4: The hyperparameter setting *hp* that maximizes the acquisition function *E* is evaluated and *G*(*hp*) get updated with the newly evaluated settings.
- Step 5: Repeats steps 2–4 until the maximum number of search architectures reaches 50.
- Step 6: The model of the hyperparameter setting *hp* with lowest classification error rate will be selected as the optimal observed model.

#### Breast lesion classification

**Fig. 2:**
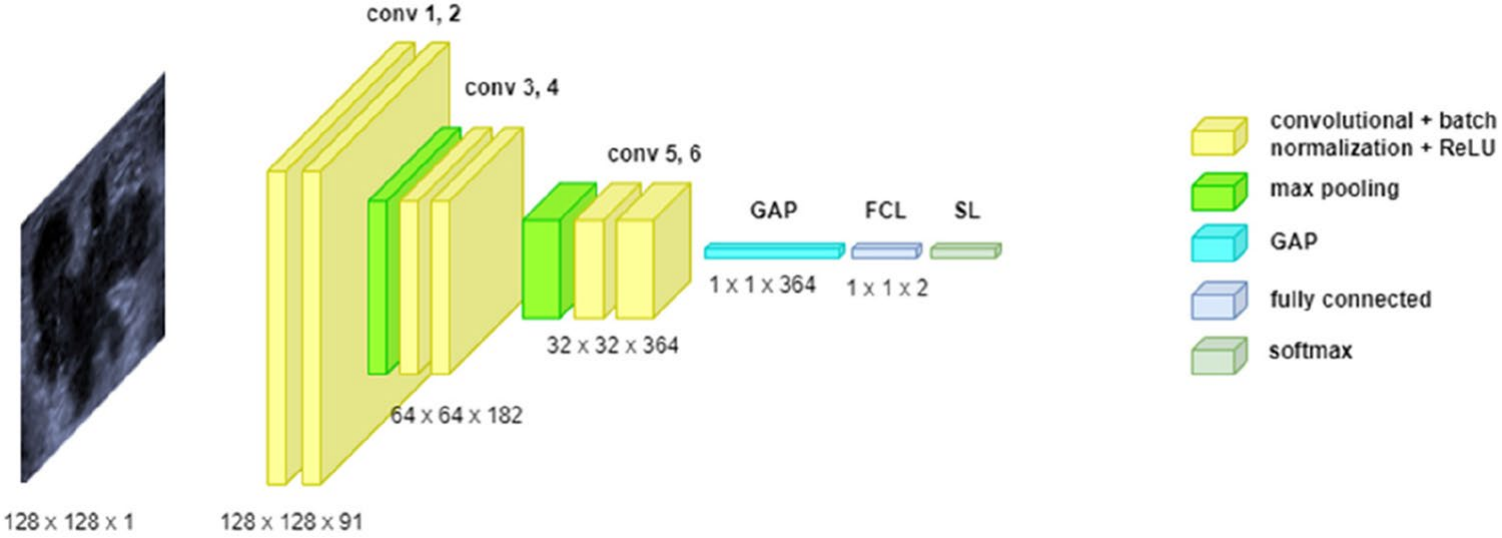
The structure of the optimal architecture

Figure 2 shows the structure of the optimal architecture resulted from Bayesian optimization search. The optimization resulted in the architectural and training parameters as follows: *hp* = {*B* = 2, *Opt* = “Adam”, *Lr* = 6.8012e − 7, *Mn* = 64, *Epo* = 50}. The architecture consists of 6 convolutional layers as shown in Fig. 2. The optimal architecture with the hyperparameter setting *hp* was used to train CNN models (BONet) for breast lesion classification. Using the internal and external datasets presented in Section 3.1, the overall average classification accuracy, specificity, sensitivity and standard deviation are reported in Section 4.

### CNN classification decision visualization

High performance of DCNN models generally accompanies with poor interpretability of model decisions due to the “black-box” nature of the models. Explaining the CNN decision of lesion recognition in 2D ultrasound images is crucial for the acceptance of CAD systems in the clinics. Saliency maps that capture the image regions contributing to the decision can be exploited to assist the understanding of the model classification output. In this study, we adopt EGrad-CAM [26] and Ablation-CAM [27] methods towards interpreting DCNN decisions. EGrad-CAM uses entropy for feature map selections prior to the saliency maps generation. We also use EGrad-CAM to compare the number of feature maps in the final convolutional layer that have either no contributions or similar characteristics for different DCNN models presented in Section 4.7. Ablation-CAM [27] removes each feature map from the final convolutional layer in an iterative fashion and then assesses whether the prediction class remains unchanged. We analyze the visualization outputs of the following layers: “relu5_4” of BNet, “inception_5b-output” of GNet, “fire9-concat” of SqNet, “res5c_relu” of RNet, “conv5_block32_concat” of DsNet, “conv_7b_ac” of IncResNet, and “conv_6” of BONet and their contributions to the final decision. Section 4.7 presents comments from expert radiologists on the highlighted image regions used by each model in terms of their relevance to the domain knowledge on malignancy characteristics.

## Experiments and results

### Experiment setup

To evaluate the performances of the classification models as outlined in the previous section, several experiments using the datasets shown in Table 1 have been designed. The first experiment compares the performance of the six network models of the transfer learning based approach, i.e., BNet, GNet, SqNet, DsNet, RsNet, and IncReNet. The second experiment evaluates the performance of our optimized network model BONet, which is then compared against another optimized ENAS model [20]. The third experiment compares the generalization errors made by all models through the differences between the accuracies of the internal tests and those of the external tests. The accuracies of all models against the number of layers, number of learning parameters, the training time, and model size are then compared in the fourth experiment. The fifth experiment further compares the performance of all the CNN models with three expert radiologists using the external datasets D and E. Besides, while testing the model performances, we also identify the image regions contributing to the classification decisions using the visualization methods mentioned in Section 3.4 in the final experiment.

For all the experiments, 1882 RoI images of 870 malignant and 1012 benign lesions from datasets A, B, and C were used for developing (i.e., training and internal testing) the models. To determine the classification accuracy for the internal testing, tenfold stratified cross validation was applied, i.e., at each iteration, the images were split, at the same ratio of benign and malignant lesions in each fold, into training examples (90%) and testing examples (10%). Among the training examples for each iteration, 10% of them were allocated as validation examples. To enlarge the training and validation dataset, we applied the data augmentation methods presented in Section 3.1 for each RoI image. These methods generated 4 additional images from each original RoI image resulting in 8469 RoI images for training and validation and 188 original RoI images for testing for each fold. The input image size is rescaled to w × h × d using bicubic interpolation according to the requirements of the input layer of a specific architecture. All experiments were run on a workstation with CPU: Intel® Xeon® Gold 5122 Processor 3.6G; GPU: Quadro GV100 32G; and 256 GB RAM.

### Breast lesion classification using DCNN models with transfer learning

Table 2 summarizes the performance of the six DCNNs in the internal tests. The table includes the averages and standard deviations (in bracket) of sensitivity, specificity, and overall accuracy across the 10 folds as well as the test accuracy of the best performing model selected from the cross validation. The selection is based on the accuracy level and the balance between sensitivity and specificity.

**Table 2:** Internal test sensitivity, specificity, and accuracy of all transfer learning based approaches

Classifier	Average performance (std) of all models			Performance of selected model		
	Sensitivity	Specificity	Accuracy	Sensitivity	Specificity	Accuracy
BNet	84.90% (4.3%)	**86.55% (2.4%)**	**85.71% (2.9%)**	88.37%	**89.22%**	88.83%
GNet	84.71% (3.5%)	85.33% (2.1%)	84.96% (2.1%)	**89.29%**	88.57%	**88.89%**
SqNet	84.69% (3%)	85.89% (1.7%)	85.28% (1.8%)	88.10%	87.50%	87.77%
DsNet	**85.08% (2.7%)**	84.3% (2.4%)	84.59% (2.1%)	87.81%	85.85%	86.70%
ResNet	82.19% (3.6%)	83.96% (3.1%)	83.05% (2.8%)	86.05%	87.25%	86.70%
IncResNet	83.11% (2.4%)	81.50% (2.5%)	82.10% (1.7%)	84.34%	83.81%	84.04%

Boldface indicates best performance

The table first shows that all models achieve an average overall accuracy between 82.10 and 85.71%. BNet achieved the highest average specificity and average overall accuracy while DsNet achieved the highest average sensitivity among all the models. Among the best performing models, the selected GNet achieved the highest sensitivity, the highest overall accuracy, and the second best specificity whereas the selected BNet model still shows the best specificity and the second best overall accuracy. The best models for the other networks are only marginally behind BNet and GNet.

As an external test, we further evaluated the performance of the best models on 1152 images from datasets D, E, F, and G. Table 3 summarizes the sensitivity, specificity, and accuracy of the six models. GNet achieved highest overall accuracy while SqNet and IncResNet achieved highest sensitivity and specificity, respectively. All models achieved overall accuracies between 81.34 and 82.38%, indicating generalization errors by all the models (see Section 4.4 for further discussions).

**Table 3:** External test sensitivity, specificity, and accuracy of the best models with transfer learning

Classifier	Sensitivity	Specificity	Accuracy
BNet	82.55%	81.18%	81.68%
GNet	84.91%	80.90%	**82.38%**
SqNet	**87.74%**	78.43%	81.86%
DsNet	79.01%	82.69%	81.34%
ResNet	84.67%	80.49%	82.03%
IncResNet	79.01%	**83.38%**	81.77%

Boldface indicates best performance

### Breast lesion classification using customized architectures

This section presents the evaluation results of our proposed method for automatically designing CNN architecture for breast lesion classification. The first part shows the results of the architecture search. The second part shows the classification accuracy of the model trained on the optimized architecture.

To optimize the hyperparameters of the backbone CNN architecture, Bayesian optimization on the datasets A, B, and C was performed. With the same tenfold cross validation partitions as described in Section 4.1, we used the first split, i.e., partition 1 for testing and the remaining partitions for training and validation, for the optimal architecture search. The optimizable hyperparameters and their search space (Section 3.3) were provided as inputs for the optimizer. The error rate of the test set has been used as the objective function, and 50 search iterations were performed. We identified the optimal and generic architecture as the one with the lowest testing error rate. The model of the optimal architecture achieved a classification error rate of 0.133%.

We used the optimal architecture as shown in Fig. 2 and the optimal hyperparameters to build the classification model BONet. To determine the classification error rates using our method, we used the same tenfold cross validation partitions. Table 4 shows the performance of our models in comparison with the state-of-the-art ENAS models. BONet outperformed ENAS on both average overall accuracy by nearly 5% and the selected best model accuracy by nearly 1.5% although ENAS has a higher sensitivity. BONet also has smaller difference gap between sensitivity and specificity.

**Table 4:** Diagnostic performance of the BONet and ENAS

Classifier	Average performance (std) of all models			Selected model		
	Sensitivity	Specificity	Accuracy	Sensitivity	Specificity	Accuracy
BONet	**84.33% (3%)**	**84.13**% **(3.2**%**)**	**84.06**% **(2**%**)**	84.42%	**84.26**%	**85.19**%
ENAS	77.36% (6.6%)	80.99% (4.8%)	79.19% (2.3%)	**85.10**%	82.40%	83.70%

Boldface indicates best performance

We further evaluated the performance of the selected best BONet and ENAS models using the 1152 cases of the external datasets D, E, F, and G. Table 5 shows the sensitivity, specificity, and accuracy of the two models. Similar to the internal test results, BONet achieved the highest overall accuracy and specificity while ENAS achieved the highest sensitivity. Besides, BONet has a gap of only 2.36% between sensitivity and specificity comparing to 14.72% of ENAS.

**Table 5:** Diagnostic performance of the selected BONet and ENAS models on the external datasets

Classifier	Sensitivity	Specificity	Accuracy
BONet	81.84%	**84.20%**	**83.33%**
ENAS	**85.86%**	71.14%	76.56%

Boldface indicates best performance

### DCNN model generalization gap

**Fig. 3:**
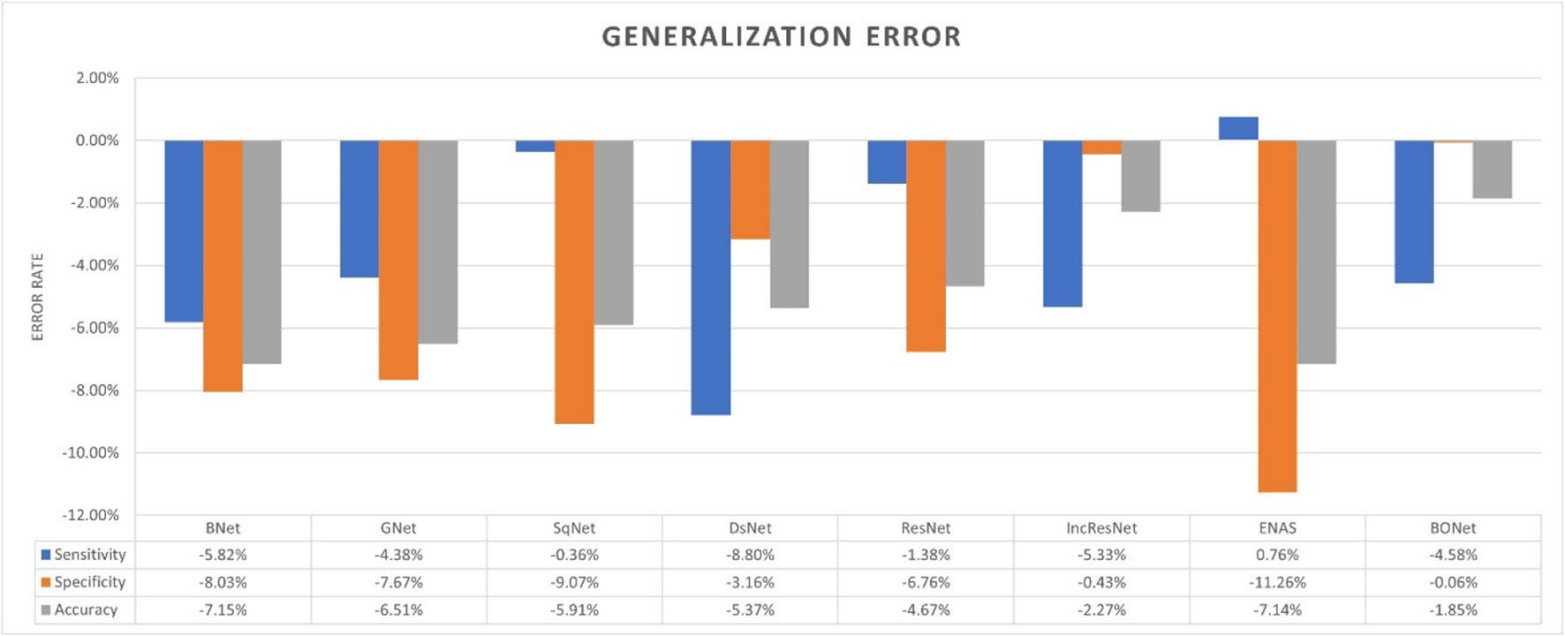
Generalization error of different CNN models

Sections 4.2 and 4.3 show that transfer learning and automatic design networks both achieved accuracy in the 80% band. This section further investigates the generalization errors of the models when tested on unseen datasets D, E, F, and G. We evaluate the generalization gap of the best performing model from each experiment in Sections 4.2 and 4.3 by measuring the difference between the average accuracy of the internal tests and the accuracy of external tests. Figure 3 shows that the gaps in the sensitivity, specificity, and overall accuracy exist across all the models particularly transfer learning based ones. BONet has the lowest generalization error with a drop in overall accuracy only by 1.85% (0.06% in sensitivity and 4.58% in specificity, respectively). BONet’s less complex architecture (see Section 4.5) partially explains the lowest generalization gap. Although ENAS generalizes well on the malignant lesion classification, it has the highest overall accuracy drop among all the models. It is worth noting that the data augmentation methods used in this study may have contributed to the reduction of model overfitting.

### DCNN model complexity

**Fig. 4:**
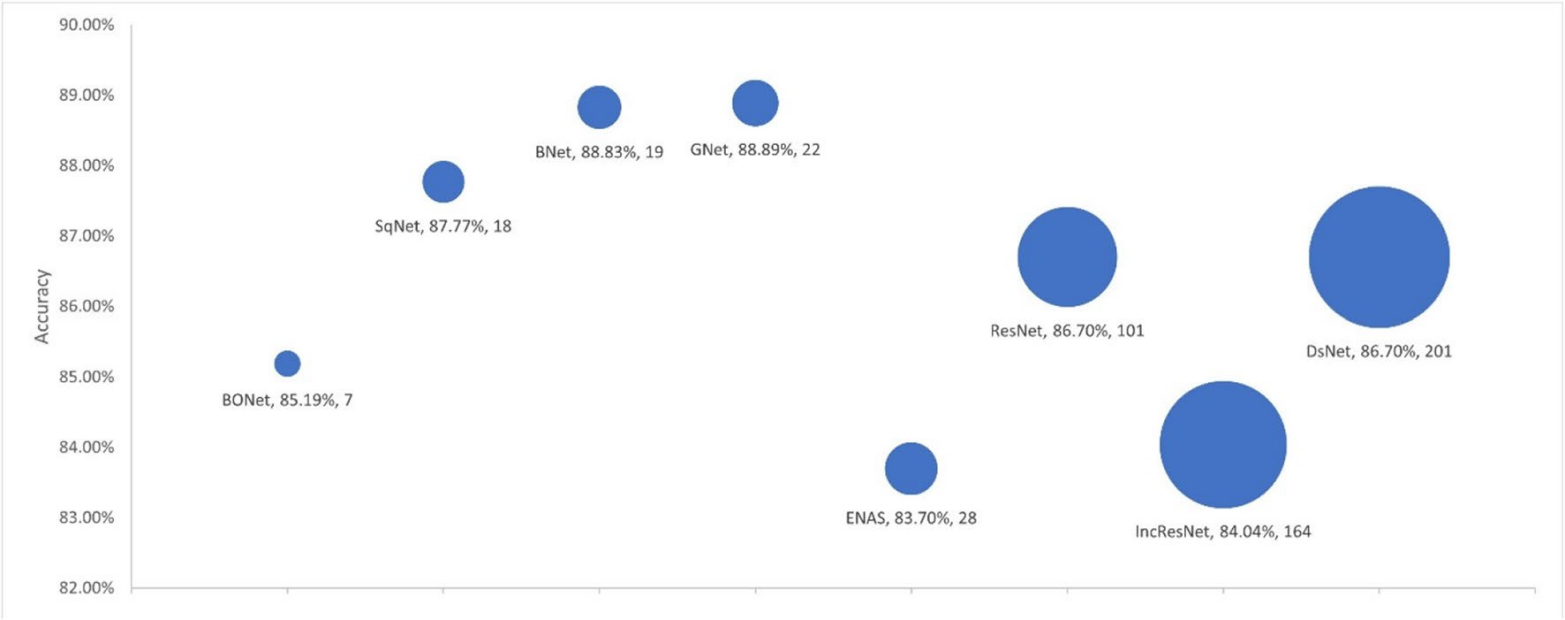
Accuracy by no. layers. The bubble size illustrates differences in the number of layers in the models

**Fig. 5:**
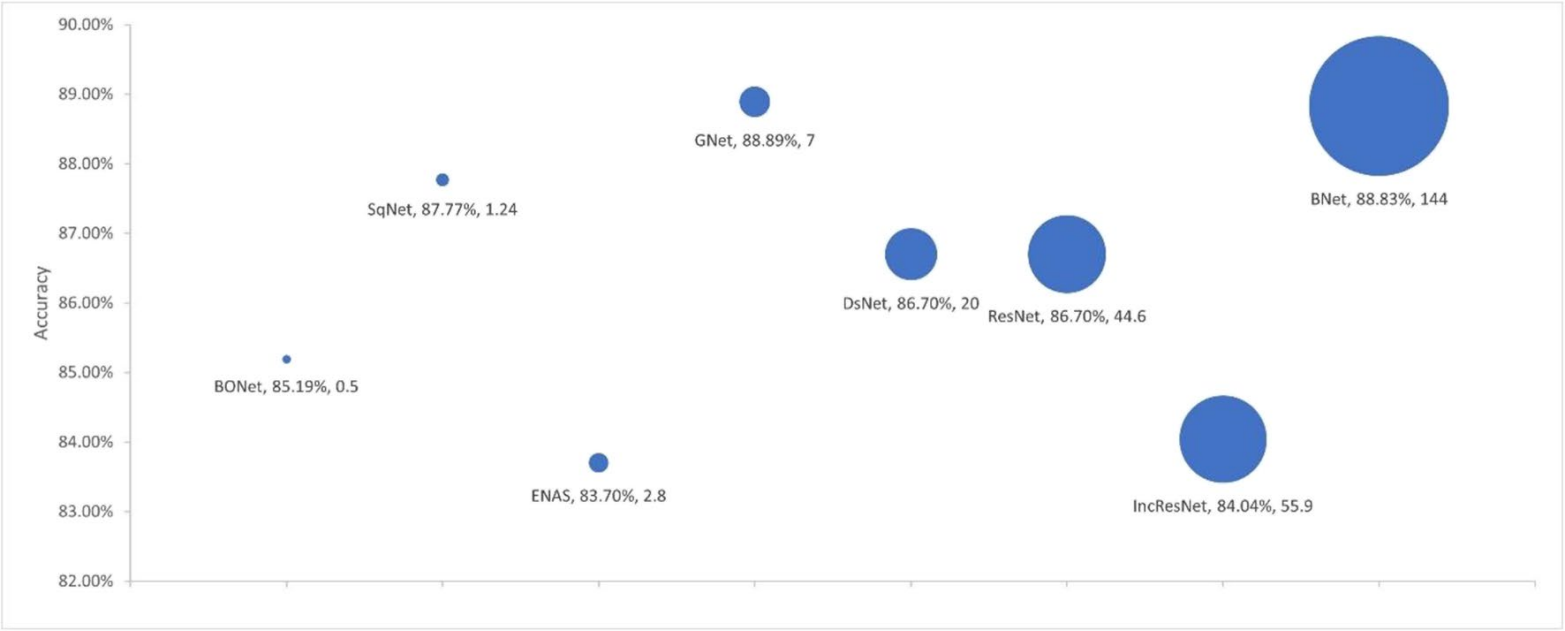
Accuracy by no. parameters. The bubble size illustrates differences in the number of parameters in millions for the models

**Fig. 6:**
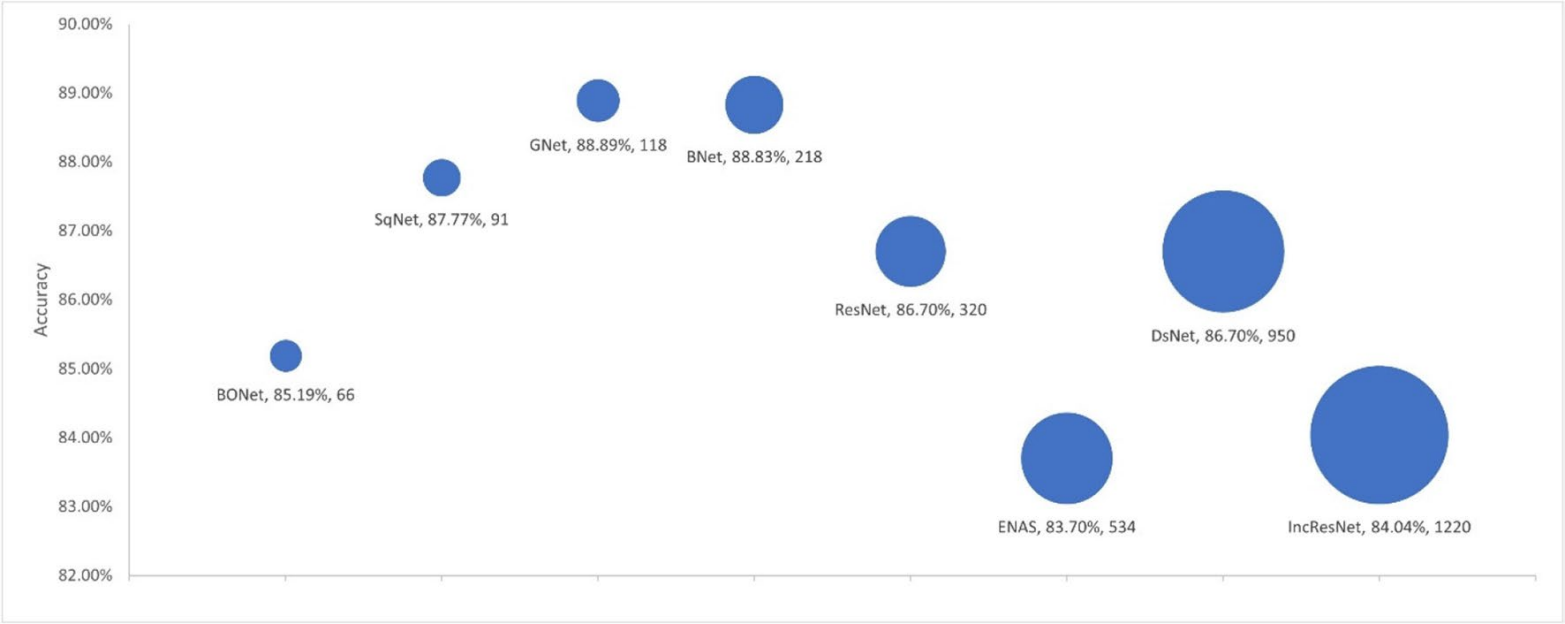
Accuracy by training time. The bubble size illustrates differences in the number of training time in minutes for the models

**Fig. 7:**
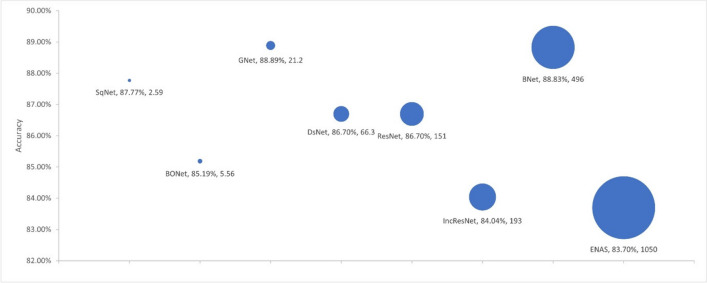
Accuracy by model size. The bubble size illustrates differences in the model size in megabytes

We used four criteria to evaluate model complexity: the number of learnable parameters, the number of layers, the model size, and the training time. We used the selected models from each experiment as presented in Sections 4.2 and 4.3 to investigate the link between the model accuracy and the model complexity. Figures 4, 5, 6, and 7 respectively show the accuracy of the models against the number of layers, the number of learnable parameters, the training time, and the model size.

The proposed BONet network has several advantages: the lowest number of layers, the lowest number of training parameters, and the least amount of training time. It is the second smallest model in size after SqNet with accuracies close to or better than other state-of-the-art networks. Such relatively small network can be installed on day-to-day workstations available in hospitals and medical centers. At the other end, complex and giant networks either have lower performances (e.g., IncResNet) or achieving a higher performance (e.g., BNet) at the price of complex structure. It is also worth noting that GNet in general maintains good balances between model complexity and accuracy.

### CNN models vs expert prediction

We further compare the classification accuracies of the DCNN models with three expert radiologists (respectively known as R1, R2, and R3) on an external data set consisting of images from datasets D and E. Table 6 shows that the overall accuracies by the radiologists are between 78.48 and 83.03%. The table also lists the performance of all the best performing CNN models. The overall accuracies of the CNN models (excluding ENAS) are higher than that of R3, matching or better than that of R2, but mostly below that of R1, except ResNet and IncResNet models that achieved marginally higher overall accuracy than all three radiologists. However, R3 still achieved the highest sensitivity (88.36%) and R2 the highest specificity (95.12%) followed by IncResNet (89.13%).

**Table 6:** Best model performance on 330 unseen test cases (datasets D and E)

Classifiers		Sensitivity	Specificity	Accuracy	|Sensitivity − Specificity|
Radiologists	R1	78.08%	86.96%	**83.03%**	**8.88%**
	R2	61.64%	**95.12%**	80.03%	33.48%
	R3	**88.36**%	70.65%	78.48%	17.71%
Transfer learning based models	BNet	81.51%	80.98%	81.21%	**0.53**%
	GNet	84.93%	81.52%	83.03%	3.41%
	SqNet	**87.67%**	75.54%	80.91%	12.13%
	DsNet	77.39%	86.41%	82.42%	9.09%
	ResNet	82.19%	85.87%	**84.24**%	3.68%
	IncResNet	78.08%	**89.13%**	**84.24**%	11.05%
Optimized network models	ENAS	86.96%	71.16%	78.16%	15.8%
	BONet	79.45%	85.33%	**82.73%**	**5.88%**

Boldface indicates best performance

The differences between the sensitivity and specificity in radiologist predictions are substantial, ranging from 8.88 to 33.48%. BNet, GNet, ResNet, and BONet have a lower difference from 0.5 to 5.88% but other CNN models also have quite large differences.

It is also worth noting the degree of agreement/disagreement among the predictions by the three radiologists as shown in Table 7. Such low degrees of agreement highlight the issue of inter-observer variability and the need for a pathology-based CAD model as a second opinion.

**Table 7:** Agreement level of three radiologists on 330 unseen test cases (datasets D and E)

Radiologist vs radiologist	Benign	Malignant	All
R1–R2	58.90%	86.41%	74.24%
R1–R3	75.34%	67.94%	71.21%
R2–R3	60.27%	70.12%	65.76%
R1–R2–R3	58.22%	67.94%	63.64%

**Table 8:** The first radiologist interpretation of the lesions and comments on the visualization of different models

Case ID	Radiologist interpretation
Case 1	This is a typical benign cyst with the following characteristics: a large proportion of anechoic echogenicity, regular edge/shape and smooth margin, and a strong enhancement at the bottom (no attenuation) Both GNet and IncResNet show areas of anechoic echo, coinciding with the benign characteristics observed from the lesion image. BONet correctly prioritizes the enhancement at the bottom and shape regularity at the top. However, the shape regularity and margin smoothness have not been noted by other DCNN models
Case 2	This lesion is malignant and invasive with the following characteristics: very irregular edge; a large proportion of unsmooth margin (small zigzags); hypoechoic for a large part of the lesion; and attenuation of echo at the bottom even without reference BNet, BONet (although more distributed), and SgNet give good indications about irregular edge and shape, and unsmooth margin on the top whereas GNet focuses more on hypoechoic echo
Case 3	Predicting the correct type of this lesion based on the US image alone is extremely difficult. The image shows almost all signs of malignancy, but the lesion can be benign. The justification for lesion benignity is the existence of a thin smooth lining on the top of the lesion between 10 and 2 o’clock positions. The appearance of such a lining is extremely rare in malignant lesions, and hence the evidence against prediction of malignancy. The presence of unclear lesion boundary and micro-calcification also make the correct diagnosis very difficult It is not surprising that the DCNN models classified the lesion incorrectly because they correctly highlight signs of malignancy. SgNet and ResNet identified the discontinuity of the smooth border for the lining at 12 o’clock. BNet and BONet highlight regions of micro-calcifications whereas DsNet and IncResNet highlight non-uniform echogenicity
Case 4	This lesion is malignant with the following characteristics: irregular shape, horizontal lesion growth, very unsmooth margin between 10 and 11 o’clock, and signs of invasiveness in the region between 2 and 4 o’clock (papillary expansions), and worse still extra-nodular expansion between 9 and 10 o’clock On the model performance, it is difficult to pinpoint areas where the models make the incorrect benign predictions. Although SqNet and RsNet seemed capturing several signs of malignancy, yet their final prediction was incorrect

**Table 9:** The second radiologist interpretation of the lesions and comments on the visualization of different models

Case ID	Radiologist interpretation
Case 1	The lesion is benign with the following characteristics: oval shape, parallel orientation, circumscribed margin, anechoic echogenicity pattern, and enhancement posterior. The predicted BI-RADS score is 2 Both BONet and GNet capture important benign signs and their correct prediction is justifiable. BONet uses the high echogenicity boundary region (fibrosis) and enhancement at the bottom whereas GNet uses the anechoic echo. GNet and IncReNet focus on the main echogenicity. The shape of the region by GNet is more accurate. However, the highlighted regions used by other CNN models are difficult to interpret and linked with the domain knowledge
Case 2	This lesion is malignant with the following characteristics: irregular shape, parallel orientation, not circumscribed (spiculated) margin, hypoechoic echogenicity pattern, and posterior features with shadowing. The predicted BI-RADS score for this lesion is 4b. However, the image quality is low. Further scans from different angles are needed to confirm the prediction BONet provides the best indication of malignancy as it captures the invasive carcinoma regions on the boundary of the lesion. The lesion boundary of this case is the most important part to make diagnostics. BNet and SqNet also capture the invasive regions but not as accurate as BONet
Case 3	The lesion type is extremely difficult to determine. From the US image only, the lesion appears as malignant with the following characteristics: irregular shape, parallel orientation, not circumscribed (angular) margin, heterogenous echogenicity pattern, and enhancement posterior features. The predicted BI-RADS score is 4c. Even if the FNA confirms this case as benign, it has a very high chance to develop into a malignant lesion The wrong predictions by the models are justifiable as all of them identified characteristics of malignancy such as echogenicity pattern. In addition, most of the models seem confusing fibrosis with calcification. IncResNet appears less confused with malignant signs
Case 4	The type of this lesion is benign with the following characteristics: irregular shape, parallel orientation, not circumscribed (angular) margin, heterogenous echogenicity pattern, and posterior features with enhancement. In addition, the case has no calcification. The predicted BI-RADS score of this lesion is 4b and the FNA is required to confirm the type GNet and DsNet use the malignant characteristics of heterogenous echogenicity regions to make the wrong decision. Similar, SqNet and IncResNet made incorrect prediction by highlighting the invasive regions on the lesion boundary (malignant characteristics)

**Fig. 8:**
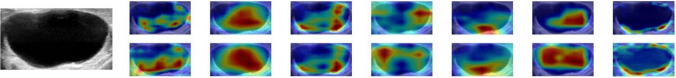
Case 1: correctly classified benign lesion by all the models (left to the right): BNet, GNet, SqNet, DsNet, RsNet, IncResNet, and BONet

**Fig. 9:**
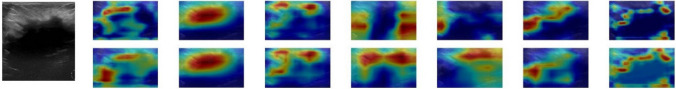
Case 2: correctly classified malignant lesion by all the models (left to the right): BNet, GNet, SqNet, DsNet, RsNet, IncResNet, and BONet

**Fig. 10:**
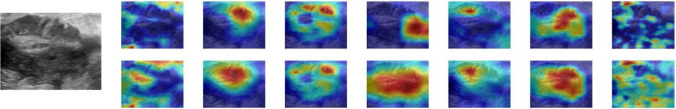
Case 3: misclassified benign lesion by the models (left to the right): BNet, GNet, SqNet, DsNet, RsNet, IncResNet, and BONet

**Fig. 11:**
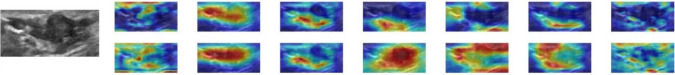
Case4: misclassified malignant lesion by the models (left to the right): BNet, GNet, SqNet, DsNet, RsNet, IncResNet, and BONet

Note that the radiologists only observed the US images by following the American College of Radiology Breast Imaging Reporting and Data System (ACR BI-RADS) [28] to describe the lesion characteristics, without information on the patient’s medical history, age, blood test, and pathology report. ACR BI-RADS is an assessment scheme that measures risk severity level of malignancy into one of seven categories (from 0 to 6). Furthermore, BI-RADS provides the US descriptors about the lesion including shape, margins, orientation, echo patterns, posterior characteristics, and calcification. We applied EGrad-CAM and Ablation-CAM to four cases classified by the CNN models: (a) a benign case correctly classified by all, (b) a malignant case correctly classified by all, (c) a benign case misclassified by all, and (d) a malignant case misclassified by all. Figures 8, 9, 10, and 11 show the visualization outputs for each case, where the top row maps are created by EGrad-CAM, and the bottom row maps by Ablation-CAM. The red color in the heatmap indicates the regions with high contribution towards the model decision while the blue color indicates regions with little contribution. Tables 8 and 9 detail the comments made by the radiologists. Both radiologists further commented that Ablation-CAM generally produces a better visualization than EGrad-CAM

### Towards understanding decisions by the CNN models

This section is intended to identify the importance score of each pixel in the lesion image for the CNN classification decision for benignity or malignancy. To link the image regions used by each model with relevant domain knowledge, we asked two of the three radiologists to explain their own diagnostic decisions and then comment on the image regions used by each model for making their decisions.

We further used EGrad-CAM to count the number of feature maps in the final layer that have no contribution to the CNN decisions. Table 10 shows the ratio of feature maps with zero entropy. The table reveals that many feature maps (from 100 to 392) in BNet and GNet have no contribution to the classification decisions. Other transfer learning based networks (except DsNet) have also similar issue. These networks were originally designed and trained on natural images. Some of their learnt features therefore have little effect in the model decisions for ultrasound image classifications of breast lesions. On the contrary, approximately, all feature maps in the final layer of our BONet model have contributed to the model decisions. In other words, the optimal CNN model (BONet) for classifying breast lesion has a thin network where most feature maps carry information for the classification decisions.

**Table 10:** Ratio of feature maps with no contribution to the model classification decision

Model (number of feature maps)	Ratio of feature maps of 0 entropy			
	Case 1	Case 2	Case 3	Case 4
BNet (512)	20.33%	34.96%	47.66%	19.53%
GNet (1024)	23.15%	38.28%	17.87%	31.45%
SqNet (512)	12.50%	13.28%	9.18%	12.70%
DsNet (1920)	0.00%	0.00%	0.00%	0.00%
RsNet (2048)	13.43%	8.45%	6.69%	11.08%
IncResNet (1536)	0.13%	0.26%	0.07%	0.20%
BONet (364)	0.00%	0.26%	0.00%	0.00%

## Discussions

Our experimental results show several findings. First, among the known transfer learning based DCNN models, their overall accuracies for both internal and external tests are similar despite marginal differences in sensitivity and specificity. Among them, GNet appears having a more balanced performance in terms of accuracy and model complexity. However, all transfer learning based DCNN models have generalization gaps. The automatic search based DCNN model BONet, despite its marginally lower accuracies in the internal tests than the transfer learning based models, has shown higher external test accuracy, robustness with the smallest generalization gap, balanced sensitivity and specificity, and simplicity in model composition. The performance of BONet is better than another automatic search method ENAS on most indicators, showing the promises of the Bayesian optimization approach for DCNN model designs for breast lesion classification in US images.

The saliency maps analysis using EGrad-CAM shows that many feature maps in the transfer learning based networks have no contribution to the classification decisions. On the other hand, most feature maps in the final layer of our BONet model carry information for the classification decisions. In this study, both EGrad-CAM and Ablation-CAM were applied on the same datasets. The study reveals differences in visualizing the regions contributing towards the model’s final decisions by the two different methods. Many factors, such as the way the weights in the feature maps are estimated, may influence these differences. Investigating the variations in the visualization by different methods is part of our future work.

## Conclusion

In this paper, we presented an evaluation of a selection of transfer learning based CNN models for breast lesion classification in US images. The evaluation shows that although there are greater differences in sensitivity and specificity all the models have comparable overall accuracies despite their architectural differences. We also presented an optimized architecture using Bayesian optimization and compared the BONet model trained on the optimized architecture with the selected transfer learning based models and an ENAS model. Experimental results show that BONet has the advantage of less complexity in network structure, least number of parameters, least amount of generalization gap with comparable level of performance, and that GNet strikes a better balance among all factors vs accuracy levels among all models. Overall, the results show the potentials of automatic architecture search in building an effective model for the intended purpose. Comparisons with three experienced radiologists demonstrated that the CNN models at least match or even outperform those of radiologists based on US images alone. The paper made a serious attempt, with the assistance of the subject specialists, to examine the link between DCNN model decisions and regions of US images for supporting the decisions using two visualization methods. The findings of this paper may lead to possible future work in several areas such as ensemble of DCNN models, expanding search space for better performing BONet, developing more effective visualization methods, and strengthening links with domain knowledge on cancer signs.
