# Correction to: Classification of breast lesions in ultrasound images using deep convolutional neural networks: transfer learning versus automatic architecture design

**DOI:** 10.1007/s11517-023-02945-5

**Published:** 2023-10-16

**Authors:** Alaa AlZoubi, Feng Lu, Yicheng Zhu, Tao Ying, Mohmmed Ahmed, Hongbo Du

**Affiliations:** 1 School of Computing and Engineering, University of Derby, Derby, DE22 3AW UK; 2 Department of Ultrasound, Shuguang Hospital affiliated to Shanghai University of Traditional Chinese Medicine, Shanghai, China; 3 Department of Ultrasound, Pudong New Area People’s, Hospital affiliated to Shanghai University of Medicine and Health Sciences, Shanghai, 201200 China; 4 Department of Ultrasound, Sixth People’s Hospital, Shanghai, China; 5 School of Computing, The University of Buckingham, Buckingham, MK18 1EG UK

**Correction to** **:** **Medical & Biological Engineering & Computing**

**https://doi.org/10.1007/s11517-023-02922-y**

The original version of this article unfortunately contained a mistake.

Figure 4 image was inadvertently copied and pasted as Figure 5 image. The correct image is shown here.

**Figure 5:**
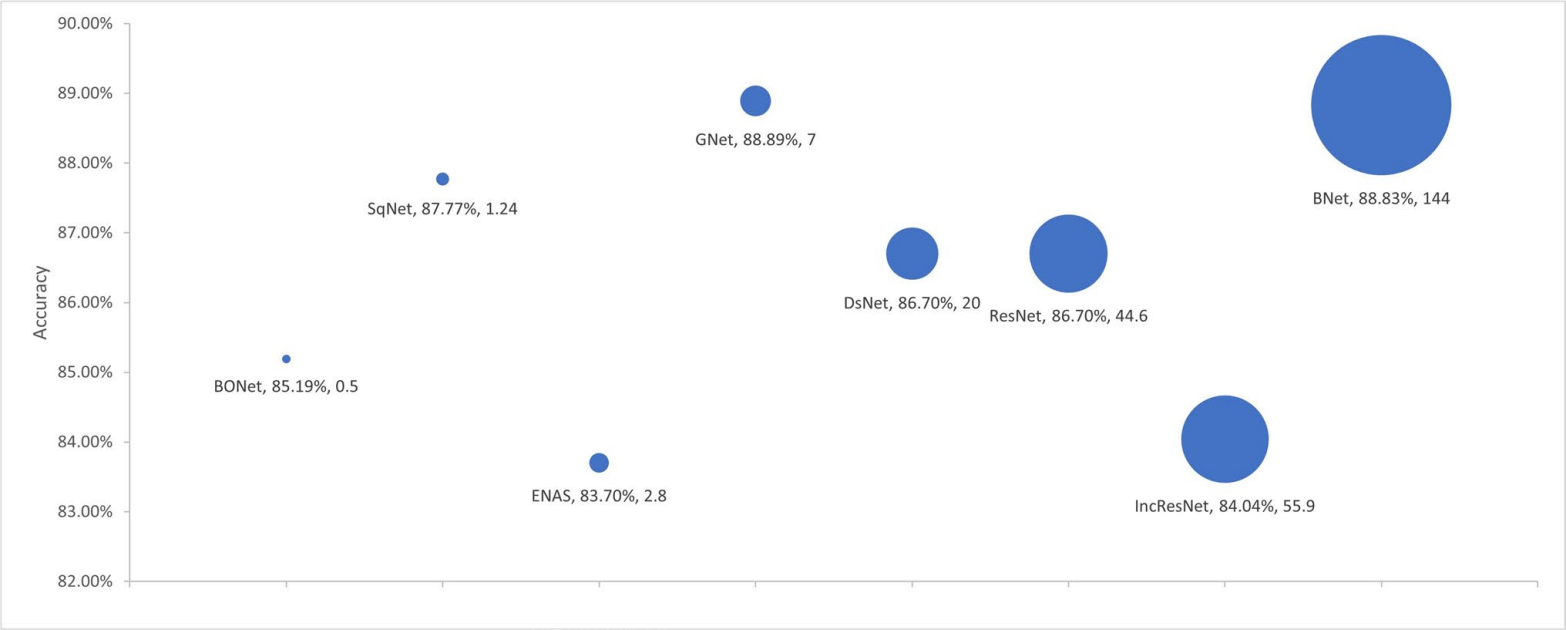
Accuracy by No. Parameters. The Bubble Size Illustrates Differences in the Number of Parameters in Millions for the Models

